# Unmet Expectations: Life Scientists’ Views on Biorisk and Responsibility

**DOI:** 10.1089/apb.2024.0052

**Published:** 2025-06-05

**Authors:** Daniel Greene, David A. Relman, Megan J. Palmer

**Affiliations:** ^1^ Center for International Security and Cooperation, Stanford University, Stanford, California, USA.; ^2^ Deloitte Consulting LLP, New York, New York, USA.; ^3^ Department of Medicine and Department of Microbiology & Immunology, Stanford University School of Medicine, Stanford, California, USA.; ^4^ Infectious Diseases Section, Veterans Affairs Palo Alto Health Care System, Palo Alto, California, USA.

**Keywords:** biorisk management, biosafety, biosecurity, dual-use, survey, proactive

## Abstract

United States and global guidance documents and codes of conduct urge life scientists to practice a “culture of responsibility” by proactively managing the potential biosafety, biosecurity, and dual-use information risks of their work. However, research suggests that many life scientists are unfamiliar with or disengaged from aspects of biorisk management. To better understand life scientists’ beliefs and attitudes about biorisk management, we conducted a survey with 127 researchers at a prestigious U.S. university who directly manipulate DNA or RNA in living organisms, cells, and/or viruses. We found that while participants were broadly positive about their efforts to address risks and expressed a sense of responsibility to do so, most failed to meet the expectations that they hold for how often a typical scientist in their research community should consider biosafety, biosecurity, or dual-use information risks. Faculty were more likely to meet their expectations than non-faculty, and all participants were more likely to meet their expectations considering biosafety risks compared with biosecurity or dual-use information risks. Most non-faculty said that they have “never or almost never” considered the risk of deliberate misuse or information release with their lab. Outside of mandatory biosafety training, few had received formal education about biorisks or discussed them at lab meetings. Career incentives and the logistical burdens of biorisk management were noted as reasons for disengagement. Our results suggest that by their own standards, U.S. life scientists have significant room for improvement in their capacity for proactive biorisk management, particularly regarding biosecurity and dual-use information risks.

## Introduction

This article summarizes the results of a survey of life scientists at a major academic research institution about their perceived responsibilities for biorisk management. Following the World Health Organization (WHO) Global Guidance Framework for the Responsible Use of the Life Sciences, we define “biorisk management” as the effort to assess and/or mitigate any of the following three types of risks:^1^

• Biosafety risks: risks of inadvertent release or unintentional exposure to biological agents or biological material from a laboratory setting.
• Biosecurity risks: risks of someone inside or outside of a lab intentionally making, modifying, or using biological material from the lab to cause harm to others.
• Dual-use information risks: risks of publishing or otherwise sharing information from research that makes it easier for others to cause harm by accidentally or deliberately misusing biological agents.

Life scientists play a key role in biorisk management. U.S. and global guidance documents and international codes of conduct emphasize that life scientists must practice a “culture of responsibility,” proactively managing the potential risks of their work by following rules in the absence of external supervision and taking action as needed when rules do not yet exist.^1–9^ In practice, proactive biorisk management often involves maintaining strong workplace norms of monitoring for risks and changing work practices or seeking out external help when issues arise.^6,10^

However, little is known about life scientists’ willingness to proactively manage biosafety, biosecurity, or dual-use information risks. Studies of workplace safety have found that safety behaviors are influenced by workers’ perceptions of the costs and benefits of those behaviors and local social norms around safety.^11^ Many life scientists perceive biosafety as an unavoidable cost of work in comparison to their intrinsic interests in science and their career pressures to “publish or perish.”^12–15^ Surveys and interviews with life scientists suggest that many feel apathetic about lab safety and fail to report lab accidents.^16–20^ There is little research on life scientists’ motivation to be vigilant about biosecurity risks, but recent studies of cybersecurity culture and practices also find that perceived costs, benefits, and social norms play a similarly important role.^21–24^ Finally, many life scientists are unaware of dual-use issues in general, construe dual-use information concerns narrowly, resist viewing their own work as dual-use, and have conflicting opinions on the value of attempts to constrain potentially dual-use information.^25–27^

A sustained program of applied social science research is needed to promote proactive biorisk management among life scientists. With a better understanding of how life scientists think and feel about biorisk management, it might be possible to redesign training, onboarding programs, and incentive structures accordingly. To this end, we conducted a survey with 127 life scientists at a large R1 research academic institution in the United States to learn more about their beliefs and attitudes about biorisk management. This institution is categorized as “R1” by the Carnegie Classification of Institutions of Higher Education, indicating that it expends over $50 million annually on research and grants over 70 research doctorates. Participants were randomly assigned to one of several survey branches to answer questions about their willingness and ability to consider and address biorisks that might arise in their work.

We aim to build on existing research in several ways. Our primary contribution is to ask life scientists how often they believe a typical scientist in their field should consider various types of risk and to compare their answers to their own self-reported behavior. This comparison allows us to evaluate life scientists on the normative standards of risk management that they set for their own fields. Our survey design also allows us to directly compare three types of risk (biosafety, biosecurity, and dual-use information risk) in one study, while previous studies have largely kept them separate, and little prior research has directly investigated life scientists’ considerations of deliberate misuse or information release.^3,18,26^ In keeping with our focus on proactive biorisk management, we also asked scientists about their own perceived responsibilities for proactive behavior as opposed to their support for top-down policies.^27^

Finally, we asked a random subset of participants about their awareness of various potential targets of harm from biorisks. Prior research suggests that many life scientists have never considered the dual-use potential of their research.^25,26^ One possible reason for this is that they are not aware of certain targets of harm, such as agricultural resources or material goods, that are included in the U.S. government’s definition of dual-use research of concern (DURC) but are less widely recognized than public health concerns.^28^

## Materials and Methods

### Survey Design

In order to begin the survey, participants must have confirmed that they were over 18 years of age and were currently working as a graduate student, postdoctoral fellow, full-time staff, or faculty member in a life-science lab that involved “directly manipulating DNA or RNA in living organisms, cells, and/or viruses.” All participants answered a set of questions about their professional and demographic background, such as their academic department, age, gender, highest academic degree obtained, and years of experience with lab research.

Each participant was then randomly assigned to one of four branches of the survey. We branched the survey because we wanted to ask many questions about three different types of biorisk while keeping the survey as short as possible to ensure that scientists would participate. Branches 1–3 all used the same set of question templates, but with different types of risk inserted into the text of the question: biosafety risks (referred to in the survey as “lab accidents with biological agents”), biosecurity risks (referred to as “deliberate misuse of lab biological agents”), or dual-use information risks (referred to as “information release” or “sharing results that are misused to cause harm”), respectively.^^*^^ To ensure that participants understood our terminology, we provided a definition and example for each type of risk (see the full survey text in Supplementary Data).

The questions in Branches 1–3 covered the following general topics:

• How often do you consider [risk] with any lab’s work?
• How often do you consider [risk] with your own lab’s work?
• How much potential for [risk] does your lab have?
• How effective is your lab at handling [risk]?
• Do you see handling [risk] as your job, officially and/or unofficially?
• How much of a burden is it for you to manage [risk], and how confident are you that you can manage it?
• Have you had training on [risk]?
• Can life scientists realistically reduce [risk] much more as a whole?
• How often does [risk] come up at lab meetings?
• How do career incentives affect engagement with [risk]?
• How often do other researchers in your subfield consider [risk]?
• Where do you get information about other researchers’ practices?
• How often should other researchers in your subfield consider [risk]?

Branch 4 asked a different set of questions on additional topics related to risks associated with information release. We were less certain about the value of the questions in Branch 4 and wanted to ensure sufficient sample size in Branches 1–3, so we weighted the randomization scheme to assign proportionally more participants to Branches 1–3 (a ratio of 3/11 participants for each) and fewer to Branch 4 (2/11). Branch 4 first asked participants about which potential targets of harm from misused life-science research they had ever considered before, such as human health, agriculture, or physical materials. It then asked participants on a four-question scale about whether they believed that there is any conceivable life-science research that is not worth pursuing because of the risks of sharing results that are misused to cause harm. (We chose not to report results for this scale because the scale showed a low Cohen’s kappa coefficient, suggesting that its individual questions were not sufficiently clear.)

We also asked two attention-check questions and timed participants to identify people who were completing the survey unrealistically fast. Finally, we asked all participants for open-response reflections on the survey itself, and all participants were also provided with a link to report lab safety concerns to their Environmental Health and Safety department. If they passed the attention-check questions and took at least 4 min to complete the survey, they were able to continue to a post-survey where they could provide their email addresses to receive $20 gift cards without their email being linkable to their previous survey responses. See Supplementary Data for a complete set of survey questions.

### Recruitment, Participation, and Data Cleaning

We conducted our survey at a U.S.-based R1 research university known for high-quality life sciences research. Our study design was approved by the university’s institutional review board (IRB). After developing an outline of the survey topics, we presented our plan at a meeting of the chairs of several life-science departments and secured their approval to email their graduate students, postdocs, staff, and faculty with an invitation to participate. For purposes of anonymity, we will not provide the exact names of participating departments. We collected the email addresses of potential participants by scraping department websites. We then piloted the survey questions with a handful of graduate students, postdocs, and faculty to make the questions as clear as possible and to minimize any misperceptions that participants might be identified or face administrative consequences for their answers.

We emailed participating departments an invitation with a survey link in January 2022 and collected responses through February 2022. Emails were sent to entire department pools and were auto-filled with participants’ names, but otherwise were not personalized. We expected that faculty would be less likely to take the survey than non-faculty because of their other time commitments, so we prioritized faculty outreach and released the survey to them first to ensure the highest possible rate of faculty participation. See Supplementary Data for the text of a survey outreach email.

We contacted 347 people and received 183 responses for a response rate of 53%. We analyzed the data in R, cutting 56 participants (31% of the raw data) who finished the survey in under 4 min (the median time to complete was about 8 min) or failed either of the two attention-check questions. We retained any responses that passed the above checks but did not finish the survey. In total, we analyzed responses from 127 participants across all four branches.

## Results

Thirty-five participants were assigned to the survey branch containing questions about lab-accident risks, 37 about deliberate-misuse risks, 33 about information-release risks, and 22 about additional questions regarding information release. Our analytic power within any single survey branch is relatively limited because we only retained 127 total responses for analysis and because participants were randomly split into four branches. For these reasons, we focus primarily on simple descriptive statistics and visualizations of raw data to illustrate general trends in our sample. In addition, we refer to “participants” as a single group in our findings, but response rates for each question vary slightly depending on the number of participants who were randomly assigned to a given survey branch and who chose to answer or skip any given question. Finally, in all graphs, faculty responses are displayed in darker shades and non-faculty in lighter shades.

### Participant Backgrounds and Nature of Work

Participants were postdocs (*n* = 47), graduate students (*n* = 33), faculty (*n* = 27), or full-time staff (*n* = 3). Participants were able to check off one or more subfields applicable to their work from an extensive list (see Supplementary Data); the most common were molecular biology (51 responses), biochemistry (50), and cell biology (47). Most participants were in their 20s and 30s (median age 33), and with four exceptions, all participants over 40 were faculty and vice-versa. Most had between 8 and 15 years of lab experience (median 10 years). About 13% of participants had served on a biosafety committee. We also asked participants to report the extent to which they saw their research as more basic (closer to a “1”) or applied (a “7”) on a 7-point scale and split responses into two bins (counting the midpoint as “more applied”). Most saw their work as more basic (*n* = 69) than applied (*n* = 32).

In general, participants worked in environments with relatively low-risk biological agents and procedures. Almost all participants worked in Biosafety Level 2+ or lower (BSL-1 = 17 participants, BSL-2 = 57, BSL-2+ = 24, BSL-3 = 4, BSL-4 = 0). Thirty-two participants worked in labs that only had Risk Group 1 organisms, 54 had some Risk Group 2, and 10 had some Risk Group 3. A total of 15 out of 96 participants worked in labs with aerosol-transmitted pathogens, and 35 out of 98 worked in labs with bloodborne pathogens.

## Considering Risks

### Self-Assessments of Potential for Harm from Risks

We asked participants about the scale of possible harm in their lab from different types of risk if they were not being careful, on a subjective 5-point scale from “no harm at all” to “a great deal of harm” following guidelines for unipolar scales.^29^ A total of 71 out of 91 responses were either “no harm at all” or “a small amount of harm,” while the remaining 20 participants provided higher estimates of potential harm, mostly from deliberate misuse and information release (see Supplementary Data).

### Overall Consideration of Risks

We asked participants how often, if ever, they consider the possibility that their lab could have either a lab accident, a deliberate-misuse incident, or an instance of dual-use information release, depending on their assigned survey branch. We also asked them how often, if ever, they think that a typical life scientist in their research community *should* consider the risk of the same three types of risk. Both questions had the same response options so that their responses could be compared, ranging from “never or almost never” to “daily.” Our analytic approach is to compare participants’ reports of their own behavior with what they expect of a typical life scientist as a standard of evaluation. For brevity, we will refer below to participants meeting, exceeding, or failing to “meet their expectations” if they self-report considering risks as much, more, or less often than what they think a typical life scientist should do.

**Figure 1.**
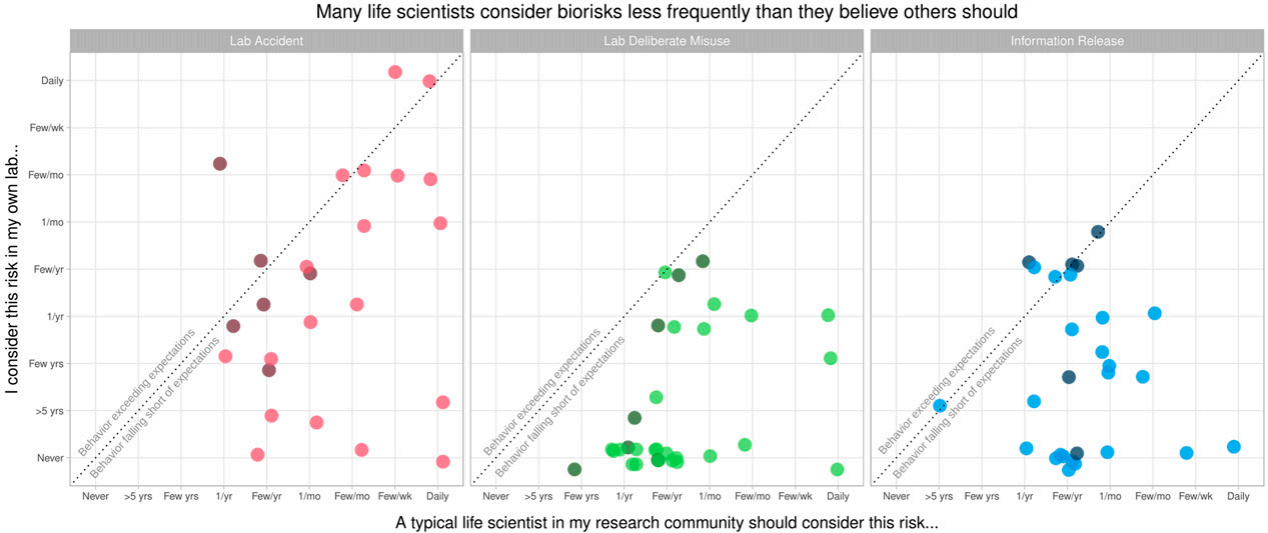
Scatter plot of faculty (dark shading) and non-faculty (light shading) responses to questions about how often a typical scientist in their research community should consider biorisks (*x*-axis) and how often they personally consider the same risks (*y*-axis). Across all three types of biorisk, most participants considered risk less frequently than they believed a typical scientist should (responses below dotted line). Faculty tended to consider risk more often than non-faculty, and participants considered lab-accident risks more frequently than deliberate-misuse and information-release risks.

Across all three types of risk, most participants failed to meet their own expectations of a typical life scientist (Figure 1). Faculty participants were more likely to meet their expectations than non-faculty, and all participants were more likely to meet their expectations with lab-accident risks than with deliberate-misuse or information-release risks. Three out of six faculty participants met or exceeded their own expectations for considering lab-accident risks, and four out of six did so for information-release risks, but only one out of seven did so for deliberate-misuse risks. In contrast, only 4 out of 19 non-faculty participants (postdocs, grad students, and staff) met or exceeded their own expectations for considering lab-accident risks, 4 out of 22 did so for information-release risks, and only 1 out of 24 did so for deliberate-misuse risks. The most common response for non-faculty was that they have *never* considered the risk of deliberate misuse or information release with their lab.

### Targets of Harm

**Figure 2.**
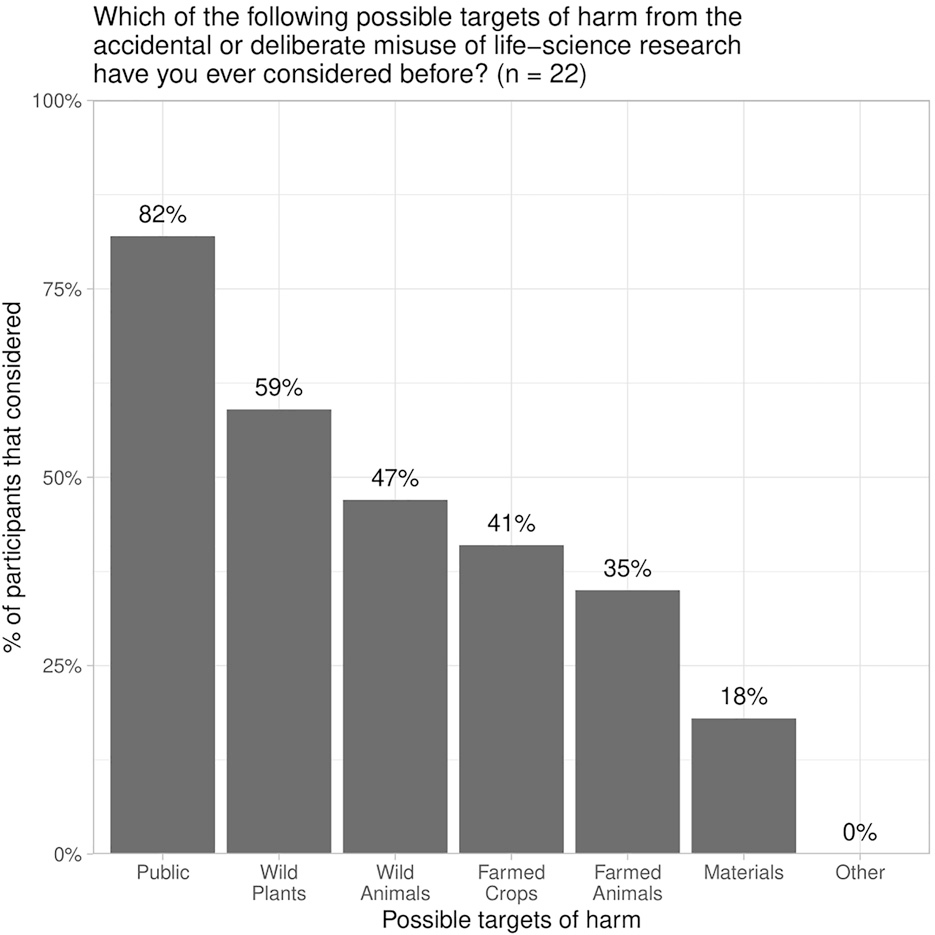
A randomly selected subset of participants (*n* = 17) were asked if they had ever considered the potential targets of harm from life-science research listed in the U.S. Government definition of dual-use research of concern (DURC).

We asked participants in one branch of the survey about whether they had ever considered a range of potential targets of harm from the accidental or deliberate misuse of life-science research. Our list of potential targets was closely inspired by the U.S. Government’s definition of DURC as threatening “public health and safety, agricultural crops and other plants, animals, the environment, materiel, or national security.”^28^ Seventeen participants responded; they were most likely to have considered potential harms to the public and much less likely to have considered wild plants or animals, agricultural crops or animals, physical materials, or other targets of harm (Figure 2).

### Training and Lab Discussions About Risks

**Figure 3.**
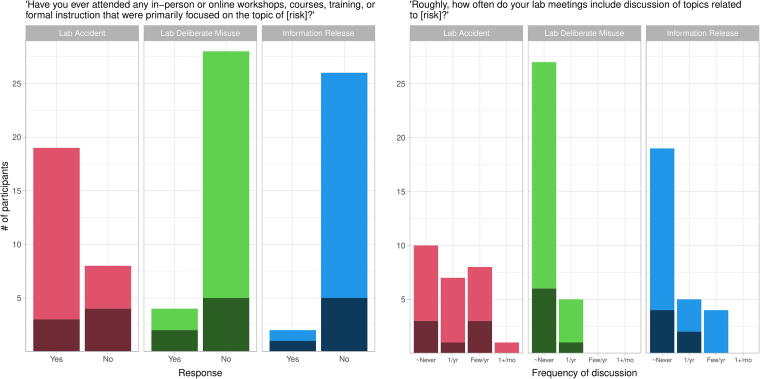
Dark shading indicates faculty responses, light shading indicates non-faculty. (Left panel) Most non-faculty participants reported receiving formal instruction about lab-accident risks. Otherwise, a majority of faculty and non-faculty participants reported not receiving formal instruction about any type of risk. (Right panel) Most participants discuss biorisks at most once per year in lab meetings, and most never discuss risks of deliberate misuse or information release. “∼Never” = “Never or almost never.”

We asked participants about whether they had “ever attended any in-person or online workshops, courses, training, or formal instruction that were primarily focused on the topic of [risk],” for all three types of risk (Figure 3, left panel). While 70% of participants reported receiving training about lab accidents, few had received training about deliberate misuse (13%) or information release (7%).

We also asked participants how often their lab meetings included discussion of topics related to each type of risk (Figure 3, right panel). For all three types, the most common response was “never or almost never”. In particular, large majorities of participants reported that their labs never or almost never discussed risks of deliberate misuse or information release in lab meetings.

## Addressing Risks

### Taking Responsibility and Lab Performance

**Figure 4.**
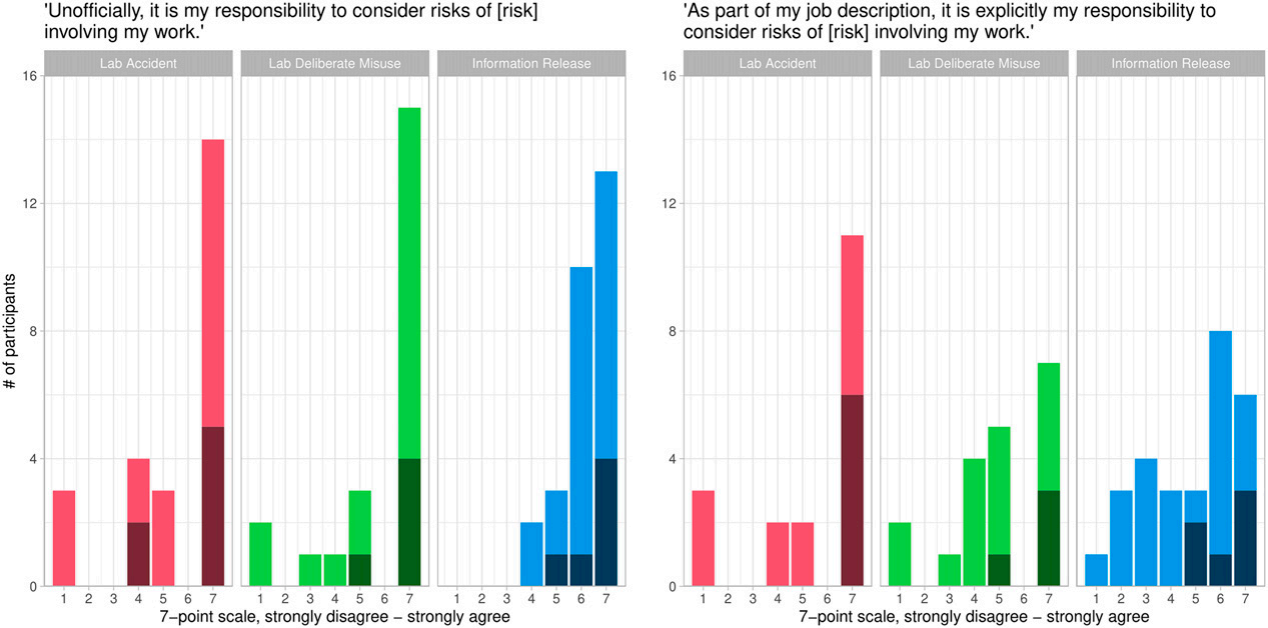
Dark shading indicates faculty responses, light shading indicates non-faculty. (Left panel) Most participants perceived an unofficial sense of responsibility to consider all three types of biorisk. (Right panel) Participants perceived varying degrees of official responsibility to consider biorisk.

We asked participants about their informal and formal responsibilities to consider the risks of their work. For each type of risk in the survey, we asked participants about their agreement or disagreement with the statement “*Unofficially, it is my responsibility to consider risks of [risk] involving my work*” on a 7-point scale. The agreement was widespread; 71% of participants at least slightly agreed about risks of lab accidents, 82% at least slightly agreed about risks of deliberate misuse, and 93% at least slightly agreed about risks of information release (Figure 4, left panel). Participants’ perceptions of their formal responsibilities were more complex. We asked them about their agreement or disagreement with the statement “As part of my job description, it is explicitly my responsibility to consider risks of [risk] involving my work.” Faculty strongly agreed with this statement across all three types of risk, while non-faculty had more mixed opinions (Figure 4, right panel).

### Burdens and Career Pressures

**Figure 5.**
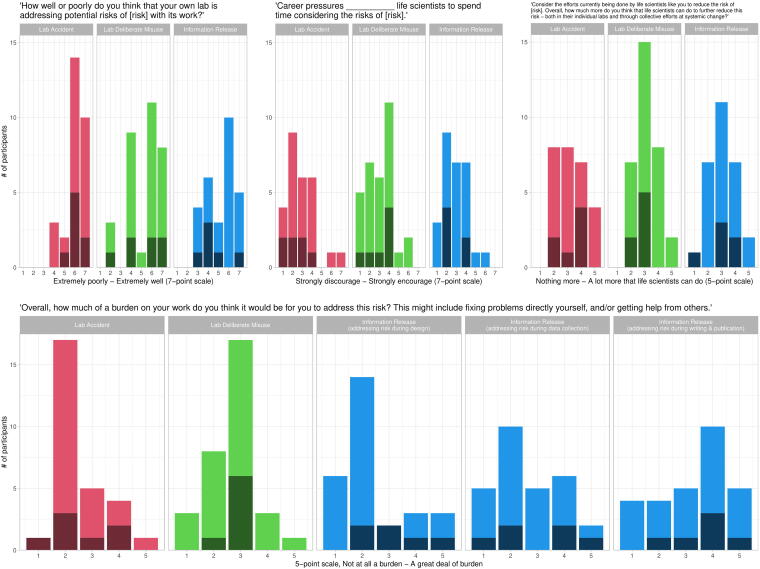
Dark shading indicates faculty responses, light shading indicates non-faculty. (Upper-left panel) Participants were mostly positive or neutral about their lab’s performance at addressing biorisks. (Upper-middle panel) Most participants perceived career pressures to either discourage life scientists from considering the risks of their work or to have no effect. (Upper-right panel) Participants generally perceived a moderate amount of potential to further reduce all three types of risk through individual or collective efforts. (Bottom panel) Most participants perceived lab-accident and deliberate-misuse risks as a mild-to-moderate burden to address. Information risks were perceived as a mild burden at the design phase of research but became more difficult to address as research progressed.

We asked participants to rate how well or poorly they thought their own lab was addressing each type of risk with its work on a 7-point scale. Participants were mostly positive or neutral about their labs’ performance, and they were more positive about addressing risks of lab accidents than about other risks (Figure 5, upper-left panel).

We also asked participants “Overall, how much of a burden on your work do you think it would be for you to address [risk]? This might include fixing problems directly yourself, and/or getting help from others.” Participants could respond on a 5-point scale from “not at all a burden” to “a great deal of burden.” We also hypothesized that risks of information release might be easier to address earlier in the lifespan of a research project, so participants in the “information release” branch of the survey were asked separately about the potential burdens of addressing information-release risks during design, data collection, and writing and publication.

Overall, lab-accident and deliberate-misuse risks were most commonly perceived as a light-to-moderate burden to address (Figure 5, bottom panel; lab-accident risks: mean = 2.54, standard deviation [SD] = 0.92; deliberate-misuse risks: mean = 2.72, SD = 0.89). Consistent with our hypothesis, participants reported that it was more and more burdensome to address the risks of information release as their research progressed. Information-release risks were perceived as the *least* burdensome of all risks on average when addressed during research design (mean = 2.39, SD = 1.26), but then became more burdensome during data collection (mean = 2.64, SD = 1.22) and became the *most* burdensome of all risks during writing/publication (mean = 3.29, SD = 1.33), a statistically significant difference (*F* = 3.68, df = 2, *p* = 0.03).

We also asked participants about the extent to which they thought that career pressures encourage or discourage life scientists from spending time considering each type of risk, on a 7-point scale (Figure 5, upper-middle panel). Only seven participants believed that career pressures encouraged life scientists to consider any risks, and most felt that career pressures discouraged them from considering risks (lab accidents: mean = 2.85, SD = 1.46; deliberate misuse: mean = 3.06, SD = 1.39; information release: mean = 2.89, SD = 1.23).

### Room to Improve

Finally, we asked participants to “Consider the efforts currently being done by life scientists like you to reduce the risk of [risk]. Overall, how much more do you think that life scientists can do to further reduce this risk—both in their individual labs and through collective efforts at systemic change?” They answered on a 5-point scale, from “nothing more that life scientists can do” to “a lot more that life scientists can do.” Almost all participants thought that something more could be done to further reduce risks; their answers centered around a “moderate” amount (Figure 5, upper-right panel; lab accidents: mean = 3.26, SD = 1.06; deliberate misuse: mean = 3.16, SD = 0.85; information release: mean = 3.07, SD = 0.98).

## Discussion

### Summary

To better understand life scientists’ willingness and ability to proactively manage biorisks, we conducted a survey with 127 researchers at a prestigious U.S. university who directly manipulate DNA or RNA in living organisms, cells, and/or viruses. Participants were a mix of grad students, postdocs, full-time staff, and faculty. They almost entirely worked in BSL-1, BSL-2, or BSL-2+, and most rated their own labs as having low or no potential for harm, with some outliers. We randomly assigned participants to one of four branches of the survey to ask a wider range of questions while limiting the survey’s length. The first three branches included a common set of questions adapted for each of three types of risk (lab accidents, deliberate misuse of lab materials, and sharing information that could be misused to cause harm). The fourth branch was assigned to have fewer participants and included a different set of questions about possible targets of harm from life-science research.

Overall, most participants considered all three types of risk less frequently than they thought a typical life scientist in their research community should. Faculty considered risks more frequently than did non-faculty, and all participants considered biosafety risks more frequently than other risks; most non-faculty participants “never or almost never” considered risks of deliberate misuse or information release. In a smaller subsample of participants, 82% had previously considered risks of harm to the public but were much less likely to have ever considered other possible targets of harm, such as wild plants or animals, agricultural plants and animals, or materials. Most participants had received “in-person or online workshops, courses, training, or formal instruction” related to lab accidents, but few had received any training related to deliberate misuse or information release. Finally, participants were most likely to “never or almost never” discuss any of the three types of risks at lab meetings, which are a key setting for labs to communicate their own social norms about scientific practice.^30^

In contrast to their efforts to consider risks, participants were broadly positive about their efforts to address risks. They felt varying degrees of responsibility to address risks depending on their position and the type of risk, but they generally reported a strong sense of informal responsibility to manage all three types. Most thought that their labs addressed potential risks well, though they were more confident about lab accidents than about other risks, and a few participants thought that their lab handled deliberate-misuse risks poorly. They reported that addressing lab-accident and deliberate-misuse risks was mostly a mild-to-moderate burden and that information-release risks became increasingly difficult to address as research progressed. They broadly agreed that career pressures discouraged life scientists from considering risks, and almost all thought that more could be done individually or collectively to further reduce risks.

### Contributions to Existing Literature and Potential for Future Work

The primary contribution of this study is to compare life scientists’ perceptions of social norms of laboratory biorisk management to their own self-reported behavior. To date, there has been little study of how life scientists perceive social norms regarding biorisk management in their field, including both descriptive norms (beliefs about what people do) and injunctive norms (beliefs about what people are socially expected to do). Assessing social norms is crucial for providing a normative ground to evaluate life scientists’ biorisk management performance. In this study, participants near-uniformly agreed that a typical researcher in their field should consider all three types at least once per year, but a large fraction of them failed to consider risks this often. More should be done to create a culture of responsibility in the life sciences.

Understanding norms and how they spread is also likely to be important for changing behavior.^7^ For example, some approaches to promoting behavior change involve educating group members about the fact that they share similar injunctive norms so that they can openly coordinate with one another.^31^ In a biorisk management context, this might involve raising awareness that life scientists may not currently be considering risks as often as they would like to. Future work could assess beliefs about biorisk management at a specific institution and then test the effects of presenting results back to the institution as a driver for individual and collective change. Notably, faculty in this study appeared to consider risks more frequently than non-faculty, and they strongly endorsed a sense of responsibility for addressing risks. Faculty could potentially play a larger role in driving changes in awareness among their postdocs, students, and staff.

This study also asked participants about their perceived responsibility to manage risks themselves, while previous survey work has tended to focus on assessing life scientists’ support for external policies and oversight.^27^ Life scientists must take a proactive role in considering and addressing some risks themselves, particularly those risks that are not adequately addressed by existing regulations.^7,10^ We found that participants largely endorsed statements of personal responsibility to manage all three types of risk—an encouraging sign for efforts to promote proactive biorisk management.

Finally, this study also extends prior literature by comparing life scientists’ beliefs and attitudes about all three types of biorisk highlighted by the WHO: laboratory accidents, deliberate misuse of biological materials, and the release of information that could be misused to cause harm. Most prior work has focused on one type of biorisk rather than comparing across types. In particular, most existing survey work has been on biosafety compliance and there has been much less on risks of deliberate misuse or information release.^3,18,26^ Similarly, we asked participants about their awareness of various targets of potential harm, which have been under-studied in previous surveys and interviews.

Our study is limited by a relatively small sample size: 127 respondents total, with only 33–37 in each of the three most important branches of the survey and only 27 faculty. However, most of our findings describe positions that were endorsed by a large majority of our sample. Our invitation response rate was also 53%, so our results may have been different if a larger fraction of our target population had responded. However, we believe it is unlikely that including the opinions of non-respondents would drastically change the pessimistic aspects of our findings. Many non-respondents are likely busy or uninterested in the topic of the survey, and given prior research on attitudes about biosafety, they would likely be less engaged with biorisk management than our respondents were. Prior work on responsible research also suggests that institutional leadership play an important role in setting norms.^4,6,7^ Future work could compare how leaders and researchers differ in their perceptions of biorisk management norms in theory and practice.

This study was also conducted in a single university, but universities and non-academic labs have different formal rules and informal social norms regarding laboratory safety and would likely also differ in other areas of biorisk management.^6^ Future research should replicate and extend our findings across institutions and contextualize them with interviews or focus groups. Our results remain broadly consistent with prior findings (often qualitative or smaller sample than our own) that many scientists across institutions are apathetic about lab safety, that life scientists are not educated about deliberate-misuse or dual-use issues, and that they face career incentives to disregard biorisk management.^13,17,18,20,25,26^ Nevertheless, readers should be cautious about generalizing our particular findings across all life scientists, particularly at non-U.S. and non-academic institutions.

Finally, this study was somewhat limited by our method of operationalizing the question of when to consider risk. We provided a simple temporal scale with options spread over a wide range of time (“every few years,” “once per month,” etc.). A temporal scale allowed us to collect broad, intuitively-clear estimates of rates of behavior over time and across many types of risk in a concise survey, but it is arguably more appropriate to consider risk at points that are determined by the nature of the work being performed.^32^ For example, it may be appropriate to assess lab-accident risks when a new piece of equipment is added to the lab, to assess deliberate-misuse risks when hiring a new lab member, and to assess information-release risks when submitting a grant proposal or developing an idea for a new project.^32–34^ A more comprehensive survey might provide a list of work milestones, and for each milestone, to ask participants to estimate the fraction of times they considered different types of risk.

### Strengthening Biorisk Management While Minimizing Added Burdens

Most participants in this study expressed the opinion that they do not consider biorisks as often as they thought a typical member of their research community should. This finding suggests that by their own standards, at least some life scientists (possibly even a majority) should consider biorisks with their work more frequently.

Some life scientists may also potentially be underestimating the risks that their current work could produce dual-use information with the potential for misuse. While most of our participants reported that their work had little potential for harm, most also have only considered risks of information release less than once per year, have received no formal instruction about information release, have never considered many potential targets of harm from biorisk, rarely or never discuss the potential for creating dangerous information in lab meetings, and report that career pressures incentivize them to not consider such risks. In addition, risks of information release may not always be as obvious as other types of risk because they need not involve work with dangerous pathogens. For example, research on gene drives, studies of immune system regulation, and computer models of viral function have all been noted as having some dual-use potential.^35,36^ In a forthcoming study, experts also disagreed widely with one another about the fraction of synthetic biology research with dual-use potential.^37^ In sum, risky research is not always immediately obvious, even to those who are conducting it.

However, many life scientists are also at least somewhat concerned about the administrative burdens of evaluating their own research for risks. On average, participants rated the current burdens of laboratory biorisk management between a 2 and 3 on a 5-point scale. In an open-response comment in this study, one respondent expressed concern that “the ethos of science that has been so beneficial for humanity, to create knowledge for everyone, is in danger of being lost in mazes of senseless bureaucracy that will stifle all scientific progress.” The challenge is to manage nascent risks, with the recognition that some risks are ambiguous, while minimizing unnecessary administrative burdens.

One approach is to place a wide scope on the work that should face *some* initial consideration of risk and then use simple processes to quickly sort out most work that is not of concern. This would reduce false negatives without putting undue burden on life scientists. For example, the Netherlands Biosecurity Office developed a publicly available “Dual-Use Quickscan” checklist to help life scientists worldwide quickly consider the dual-use potential of a research project.^38^ If life scientists ran through such a checklist with all of their projects, the large majority would arguably pass with ease and only a small fraction would warrant greater attention. Scientists themselves could develop field-standard checklists in partnership with biorisk professionals, in the manner of previous collaborations on open-science publication practices.^39^ Checklists could also be effectively integrated with lab management software or similar systems to send reminders to researchers and allow for easy updating.

## Conclusions

The results of this study suggest several topics that should be included in curricula for promoting proactive biorisk management among life scientists. First, training should not only cover risks associated with lab accidents but also risks related to the deliberate misuse of materials and the release of dangerous information. Second, training should make life scientists more aware of possible targets of harm beyond human health and the natural environment, such as agricultural plants and animals. Third, training should reflect life scientists’ own collective beliefs regarding biorisk management back to them in order to strengthen social norms. In particular, training programs should summarize data conveying that life scientists (at least those in our sample) collectively support the value of training and that most feel responsible for managing the risks of their work.

Training should also clarify exactly what lab members should do to manage unfamiliar potential biorisks once they are recognized. The need for specific advice is less urgent for lab-accident risks, which are more likely to have institutional protocols, and more urgent for risks of deliberate misuse and information release. While a full discussion of the details of biorisk management is outside of the scope of this article, life scientists facing unfamiliar potential safety or security risks should likely make contact with better-informed representatives from their home institution. Training programs should ensure that scientists know whom to call and that they feel safe and comfortable doing so.

In closing, the findings in this article reveal potential gaps in many U.S. life scientists’ efforts at proactive biorisk management. However, they also suggest promising ways forward. Many faculty participants are meeting or exceeding their expectations for others to consider biorisks, and many report being willing to take personal responsibility for biorisk, but they have not received guidance on how to do so. Faculty are also an obvious channel for communicating norms of proactive biorisk management to non-faculty, who appear to consider risks less frequently. Raising awareness, providing training, building relationships with biosafety staff, and shaping norms and incentives would likely all help to promote proactive biorisk management.

## Notes

^*^

*For clarity, we restricted our definitions of risks compared with those in the WHO Global Guidance Framework.^1^ The WHO definition of biosecurity also includes “unauthorized access to or loss, theft, misuse, diversion” of biological agents, and its definition of dual-use information risks includes sharing products as well as information and also includes unintentional misuse.*
