# Supplementary material for: Unmet Expectations: Life Scientists’ Views on Biorisk and Responsibility
Source: Appl Biosaf. 2025 Jun 5;30(2):112–23. doi: 10.1089/apb.2024.0052 (PMC12179379; doi:10.1089/apb.2024.0052)
Supplement: Supplementary Data [file apb.2024.0052_supplementary_data.docx]

## Complete text of survey

### Introduction, consent form, initial questions

Thank you for agreeing to participate in the survey! Please read this whole page carefully before continuing.

We'd like to ask you for your opinions about risks that might potentially arise with life science research. Your answers are anonymous, and can help improve biosafety training at [institution] and inform life-science policy.

The survey should take about 12 minutes, and you will be paid $20 for participating.

In order to qualify for the survey, all of the following must be true:

- You are a faculty member, postdoc, grad student, staff member, or visiting scholar at [institution]. (Undergraduates are not included in this survey.)
- You participate in lab research that involves directly manipulating DNA or RNA in living organisms, cells, and/or viruses.
- You are over 18 years old.

Please answer the survey honestly, for three reasons.

1. First, it helps us to do better research. We really do want to know how you think about these issues, and your answers will help us learn.
2. Second, there are no penalties or right or wrong answers for any of these questions. They are just your opinions.
3. Finally, your individual answers are anonymous. We will collect your email address in a separate survey to send you payment, but this second survey cannot be linked to your responses to the first survey.

Also, please note that there may be checks in the survey to ensure that you are paying attention and not speeding through.

Finally, please note that this research is not related to the COVID-19 pandemic, and has been in development since before the start of the pandemic.

Thanks again. Please click forward to read a consent form and start the survey.

**Consent form**

Study Title: Mixed-Methods Investigation of Social Responsibility in the Life Sciences

[name], Protocol Director

Dear Participant: You are invited to participate in a research study that explores the opinions that people working in the life sciences hold about the potential risks and benefits of their work. If you decide to participate, you will participate in an online survey in which you may be asked to answer questions about your background, share your opinions about various topics, and/or read texts and write short responses.

In order to qualify for this study, you must be over 18 years old and currently work as a faculty member, postdoc, grad student, staff member, or visiting scholar in an academic life-science lab at [institution] that involves directly manipulating DNA or RNA in living organisms, cells, and/or viruses.

Your participation will take about 12 minutes, and you will be paid $20 for your participation via a gift card set to your email address. Beyond this, we cannot guarantee that you will receive any benefits from this study, but there are also no risks associated with participation. Your decision about whether or not to participate in this study will not be counted against you in any way. However, the knowledge gained from this study may help researchers develop better educational programs for life scientists, so we hope that you will be eager to participate. Finally, there may be periodic attention checks to ensure you are paying attention. If you fail these checks, you will not receive compensation.

Please understand that your participation is voluntary, and you have the right to withdraw your consent or stop participating at any time without any penalty or loss of benefits to which you are otherwise entitled. You also have the right to refuse to answer particular questions. Your individual privacy will be maintained in all published data resulting from the study.

If you have any questions, concerns or complaints about this research study, its procedures, risks and benefits, please contact the Protocol Director, [name], at [email] or by phone at [phone]. If you are not satisfied with how this study is being conducted, or if you have any concerns, complaints, or general questions about the research or your rights as a participant, please contact the [institution] Institutional Review Board (IRB) to speak to someone independent of the research team at [number] or toll free at [number]. You can also write to the [institution] IRB, [address].

By responding to the questions below and continuing forward, you acknowledge that you are over 18 years old, you have read the text above, and you consent to participate in the study.

Protocol approval date: 05/11/2020

What year were you born? Please write a number, like "1980". [open response]

Do you currently work in an academic life-science laboratory? [yes/no]

What position(s) do you hold in your laboratory? Please select all that apply.

- Undergraduate student
- Graduate student
- Postdoc
- Part-time staff - research/technical
- Part-time staff - management
- Full-time staff - research/technical
- Full-time staff - management
- Assistant Professor
- Associate Professor
- Professor
- Visiting Scholar
- Other: [open response]

Does your work involve directly manipulating DNA or RNA in living organisms, cells, and/or viruses?

- Yes
- No
- I don't know

What is the Biosafety Level (BSL) of your lab?

- BSL-1
- BSL-2
- BSL-2+
- BSL-3
- BSL-4
- I don’t know

Which subfield(s) of the life sciences best describes your lab? Please select all that

apply.

- Biochemistry
- Biophysics
- Structural Biology
- Bioengineering
- Synthetic Biology
- Cell Biology
- Developmental Biology
- Ecology
- Evolutionary Biology
- Genetics
- Genomics
- Bioinformatics
- Immunology
- Infectious Disease
- Microbiology
- Molecular Biology
- Virology
- Neuroscience
- Neurobiology
- Pharmacology
- Toxicology
- Environmental Health
- Physiology
- Animal Sciences
- Entomology
- Food Science and Engineering
- Systems Biology
- Other: [open response]

What is your department at [institution]? [open response]

Roughly, how often do you think about the broader potential impacts of your research on the world?

- Never
- Less than once every 5 years
- Once every few years
- Once per year
- A few times per year
- Once per month
- A few times per month
- A few times per week
- Daily

### Branches 1-3: Risk-specific questions

*[Note: the next section of the survey randomizes participants into one of four branches. Branches 1-3 use nearly-identical question structures but ask about one of three different types of risk. At the start of each branch, participants read a short description of their randomly­-assigned type, as follows:]*

### Branch 1 risk definition: Lab accidents with biological agents

**"Lab accidents with biological agents"** happen when someone makes a mistake, or something in the lab malfunctions, and a biological agent is released and causes harm to people and/or the environment. (A "biological agent" here is an organism or virus or one of its byproducts.)

Note that this doesn't count accidents from things other than biological agents, like broken glass or slips and falls.

An example of a lab accident with a biological agent: Someone accidentally blocks the vents of a biosafety cabinet and gets infected with a pathogen.

### Branch 2 risk definition: Deliberate misuse of lab biological agents

**"Deliberate misuse of lab biological agents"** happens when someone inside or outside the lab intentionally makes, modifies, or uses a biological agent from the lab to cause harm to others. (A "biological agent" here is an organism or virus or one of its byproducts.)

An example of deliberate misuse of lab biological agents: A lab staff member who wants to sicken other people deliberately cultivates and releases a pathogen that is being studied in the lab.

### Branch 3 risk definition: Sharing results that are misused to cause harm

**"Sharing results that are misused to cause harm"** happens when a life scientist publishes or otherwise shares information from research that makes it easier for other people to cause harm by accidentally or deliberately misusing biological agents.

An example of sharing results that are misused to cause harm: A life scientist publishes a paper explaining a new and easy technique for modifying a common virus. Someone else reads the paper and sees how the technique can be used to make the virus more harmful, and then uses it to create and then accidentally or deliberately release a harmful virus.

### Question structures for Branches 1-3

Thanks. This next section has some questions about a specific kind of risk that could potentially arise in life science research. Please carefully read the following definition:

[randomly-assigned definition of one of three risk types as described above]

How often, if ever, do you consider the possibility that a life-science lab (inside or outside of [institution]) could have a problem with [risk]?

- I never consider this possibility
- Less than once every 5 years
- Once every few years
- Once per year
- A few times per year
- Once per month
- A few times per month
- A few times per week
- Daily

How often, if ever, do you consider the possibility that your lab could have a problem with [risk]?

- I never consider this possibility
- Less than once every 5 years
- Once every few years
- Once per year
- A few times per year
- Once per month
- A few times per month
- A few times per week
- Daily

Hypothetically, IF your lab was not being careful at all, how much potential harm could be caused by [risk]?

- No harm at all, or almost no harm
- A slight amount of harm
- A moderate amount of harm
- A large amount of harm
- A great deal of harm

How well or poorly do you think that your own lab is addressing potential risks of [risk]?

- Extremely well
- Moderately well
- Slightly well
- Neither well nor poorly
- Slightly poorly
- Moderately poorly
- Extremely poorly

How much do you agree or disagree with the following statements?

Other people are already doing a good enough job managing the risk of [risk] with my work, so I don't really need to consider it.

- Strongly disagree
- Disagree
- Slightly disagree
- Neither agree nor disagree
- Slightly agree
- Agree
- Strongly agree

If I don't personally consider the risk of [risk] with my work, nobody else will do a good enough job managing it.

- Strongly disagree
- Disagree
- Slightly disagree
- Neither agree nor disagree
- Slightly agree
- Agree
- Strongly agree

Imagine that you spotted a potential risk of [risk] involving your lab's work, and you thought that it could be dangerous.

Overall, how much of a burden on your work do you think it would be for you to address this risk? This might include fixing problems directly yourself, and/or getting help from others.

- Not at all a burden
- A slight burden
- A moderate burden
- A large burden
- A great deal of burden

*[Note: We modified the question above slightly for Branch 3, “sharing results that are misused to cause harm”. We added the following preface: “This might come up during an early design stage of research, during hands-on data collection, or during writing and publication.” Then we asked the question above three times, asking about the burdens of addressing risk “during design”, “during data collection”, and “during writing and publication”.]*

How confident would you be in your ability to address this risk?

- Not at all confident
- Slightly confident
- Moderately confident
- Very confident
- Extremely confident

How much do you agree or disagree with the following statements?

As part of my job description, it is explicitly my responsibility to consider risks of [risk] involving my work.

- Strongly disagree
- Disagree
- Slightly disagree
- Neither agree nor disagree
- Slightly agree
- Agree
- Strongly agree

Unofficially, it is my responsibility to consider risks of [risk] involving my work.

- Strongly disagree
- Disagree
- Slightly disagree
- Neither agree nor disagree
- Slightly agree
- Agree
- Strongly agree

Have you ever attended any in-person or online workshops, courses, training, or formal instruction that were primarily focused on the topic of [risk]?

- Yes
- No
- I don't know

Consider the efforts currently being done by life scientists like you to reduce the risk of [risk]. Overall, how much more do you think that life scientists can do to further reduce this risk - both in their individual labs and through collective efforts at systemic change?

- There is nothing more that life scientists can do
- There is a little more that life scientists can do
- There is a moderate amount more that life scientists can do
- There is a lot more that life scientists can do
- There is a great deal more that life scientists can do

Roughly, how often do your lab meetings include discussion of topics related to [risk]?

- Never or almost never
- Once per year
- A few times per year
- Once per month or more

Please fill in the blank in the way that best matches your opinion: "Career pressures __________ life scientists to spend time considering the risks of [risk]."

- strongly encourage
- moderately encourage
- slightly encourage
- neither encourage nor discourage
- slightly discourage
- moderately discourage
- strongly discourage

Picture a TYPICAL life scientist that you might work with in your research community. How often, if ever, do you think that they consider the risk of [risk] in their lab?

(Note: we are asking about how often they DO consider risk, not how often they SHOULD - we will ask that in a minute.)

- They never think about this possibility
- Less than once every 5 years
- Once every few years
- Once per year
- A few times per year
- Once per month
- A few times per month
- A few times per week
- Daily

How certain are you about your answer above?

- Not at all certain
- Slightly certain
- Moderately certain
- Very certain
- Extremely certain

How did you decide your answers above? Please check all that apply.

- I know that other life scientists are required by their institutions to follow the standard rules for handling this risk
- I generally assume that other life scientists are doing a good job with handling this risk
- I visit other life scientists and directly observe how they deal with this risk
- I talk with life scientists working in other labs about how they deal with this risk
- I read about how other life scientists deal with this risk in their publications
- Other: [open response]

How often, if ever, do you think that a typical life scientist in your research community SHOULD consider the risk of [risk] in their lab?

- They should never think about this possibility
- Less than once every 5 years
- Once every few years
- Once per year
- A few times per year
- Once per month
- A few times per month
- A few times per week
- Daily

### Branch 4: Additional questions about information risks

*[Note: Some participants were randomized to Branch 4 to answer a separate set of questions about information risks unrelated to Branches 1-3. As noted in the main text of the article, not all of the questions in this Branch are reported.]*

Thanks. This next section has some questions about a specific kind of risk that could potentially arise in life science research.

Please carefully read the following page before continuing.

Some people might argue that every life-science research project creates the potential for benefits and harms, even if those benefits and harms are very unlikely.

The benefits of life-science research are familiar, such as creating beneficial technologies or products, raising awareness about important issues, or simply extending our understanding of the world.

But there might also be risks from some kinds of life-science research. In this section of the survey, we are interested in considering one particular kind of risk, which we will call **"sharing results that are misused to cause harm".**

This happens when a life scientist publishes or otherwise shares information from research that makes it easier for other people to cause harm by accidentally or deliberately misusing biological agents.

An example of sharing results that are misused to cause harm: A life scientist publishes a paper explaining a new and easy technique for modifying a common virus. Someone else reads the paper and sees how the technique can be used to make the virus more harmful, and then uses it to create and then accidentally or deliberately release a harmful virus.

There is some debate in the life sciences about whether the risk of sharing results that are misused to cause harm could ever outweigh the expected benefits of performing life-science research.

Some people argue that the risk of sharing results that are misused to cause harm virtually never outweighs the benefits of performing life-science research. Other people argue that some life-science research could create information that has so much potential for misuse that the research is not worth performing.

What do you think? Please carefully read and respond to each of the following statements. (Note that we write similar statements in different ways in order to make sure that we understand your perspective.)

The benefits of pursuing life-science research always outweigh any risk of sharing results that are misused to cause harm.

- Strongly disagree
- Disagree
- Slightly disagree
- Neither agree nor disagree
- Slightly agree
- Agree
- Strongly agree

The risk of sharing results that are misused to cause harm is never a good reason to hold back life-science research.

- Strongly disagree
- Disagree
- Slightly disagree
- Neither agree nor disagree
- Slightly agree
- Agree
- Strongly agree

Some life science research should not be pursued because of the risk of sharing results that are misused to cause harm.

- Strongly disagree
- Disagree
- Slightly disagree
- Neither agree nor disagree
- Slightly agree
- Agree
- Strongly agree

Some life-science research could create information that has so much potential for misuse that it is not worth pursuing.

- Strongly disagree
- Disagree
- Slightly disagree
- Neither agree nor disagree
- Slightly agree
- Agree
- Strongly agree

Which of the following possible targets of harm from the accidental or deliberate misuse of life-science research have you ever considered before? Please check all that apply.

- Health and safety of individual people (e.g. of lab staff)
- Health and safety of the public, or larger communities of people
- Agricultural crops and other plants
- Farmed animals
- Wild animals
- Farmed plants/crops
- Wild plants or other non-animal organisms
- Materials, equipment, and infrastructure
- Other: [open response]

### Final questions

Please choose the last option below to indicate that you are reading the survey carefully.

- Strongly agree
- Agree
- Somewhat agree
- Neither agree nor disagree
- Somewhat disagree
- Disagree
- Strongly disagree

About how many years have you worked in life-science laboratories? [open response]

Have you ever served on a biosafety committee or a similar group? [yes/no]

Do you consider your research more "basic" or more "applied"?

- Much more basic
- Moderately more basic
- Slightly more basic
- Equally basic and applied
- Slightly more applied
- Moderately more applied
- Much more applied

What is the highest Risk Group of any biological agent used in your lab?

- Risk Group 1 - Agents that are not associated with disease in healthy adult humans.
- Risk Group 2 - Agents that are associated with human disease which is rarely serious and for which preventive or therapeutic interventions are often available.
- Risk Group 3 - Agents that are associated with serious or lethal human disease for which preventive or therapeutic interventions may be available.
- Risk Group 4 - Agents that are likely to cause serious or lethal human disease for which preventive or therapeutic interventions are not usually available.
- I don't know

Does your lab work with any aerosol-transmissible pathogens? [yes/no/I don’t know]

Does your lab work with any bloodborne pathogens, or infectious microorganisms in human blood that can cause disease? [yes/no/I don’t know]

What is your gender? [male/female/other (please specify)/prefer not to say]

What is the highest level of education that you have completed? [Did not complete high school or GED; High school or GED; Undergraduate degree; Master's degree; Doctoral degree]

Were you born before 1905? [yes/no]

Thank you again for taking the time to participate. This is a pilot survey, and we are still adjusting the questions. If you have any open-ended feedback, comments about the survey, or suggested future topics to include, feel free to leave an optional note below. We read every response, and your individual responses will not be shared outside of the research team. Also, if you have any concerns about risks arising from life-science research at [institution], you can contact [institution]’s Environmental Health and Safety office at this link [link]. They accept anonymous responses. [open response]

Do you wish to receive a $20 Amazon gift card for completing this survey? If you answer yes, you will be taken to a second survey so that you can enter your email address. (Your email will not be linked to the responses in this survey.) [Yes/No]
